# Viewing Time Measures of Sexual Orientation in Samoan Cisgender Men Who Engage in Sexual Interactions with *Fa’afafine*

**DOI:** 10.1371/journal.pone.0116529

**Published:** 2015-02-13

**Authors:** Lanna J. Petterson, Barnaby J. Dixson, Anthony C. Little, Paul L. Vasey

**Affiliations:** 1 Laboratory of Comparative Sexuality, Department of Psychology, University of Lethbridge, Lethbridge, Canada; 2 Evolution & Ecology Research Centre, School of Biological, Earth and Environmental Sciences, University of New South Wales, Sydney, Australia; 3 Behaviour & Evolution Research Group, School of Natural Sciences, University of Stirling, Stirling, United Kingdom; Brock University, CANADA

## Abstract

*Androphilia* refers to attraction to adult males, whereas *gynephilia* refers to attraction to adult females. The current study employed self-report and viewing time (response time latency) measures of sexual attraction to determine the sexual orientation of Samoan cisgender men (i.e., males whose gender presentation and identity is concordant with their biological sex) who engage in sexual interactions with transgender male androphiles (known locally as *fa’afafine*) compared to: (1) Samoan cisgender men who only engage in sexual interactions with women, and (2) *fa’afafine*. As expected, both measures indicated that cisgender men who only engaged in sexual interactions with women exhibited a gynephilic pattern of sexual attraction, whereas *fa’afafine* exhibited an androphilic one. In contrast, both measures indicated that cisgender men who engaged in sexual interactions with *fa’afafine* demonstrated a bisexual pattern of sexual attraction. Most of the cisgender men who exhibited bisexual viewing times did not engage in sexual activity with both men and women indicating that the manner in which bisexual patterns of sexual attraction manifest behaviorally vary from one culture to the next.

## Introduction

In many cultures, worldwide, more than two genders are recognized beyond the binary of “man” and “woman” (the terms *male* and *female* refer to an individual’s biological sex, regardless of the individual’s gender role presentation as a boy/man, girl/woman, or otherwise). In particular, a preponderance of alternative gender roles exist cross-culturally for feminine/transgender males (*transgender* is a Western term that refers to an individual whose gender identity or gender role presentation does not match their biological sex). Examples include, but are by no means limited to, the *bissu* of Sulawesi [1], the *hijra* of India [2], the *xanith* of Oman [3], the *muxes* of Mexico [4], the *‘yan dandu* of Nigeria [5], and the *fa’afafine* of Samoa [6]. Alternative gender role categories, such as these, often mark feminine/transgender males being neither “men”, nor “women” within the context of their respective cultures. Consequently, such males are sometimes referred to in the academic literature as members of a “third gender” (e.g., [7]).

These third gender males are, almost always, exclusively *androphilic* (i.e., sexually attracted to adult males). Therefore, such males have been described in the academic literature as *transgender androphilic males* (e.g., [8]). Although they are androphilic, transgender androphilic males do not typically engage in sexual activity with each other. Rather, they engage in sexual activity with masculine, cisgender males (i.e., males whose gender presentation and identity is concordant with their biological sex) who self-identify, and are identified by others, as “men” [9].

From an emic perspective (an emic understanding of the world focuses on how people within a culture think [10]), sexual interactions between transgender androphilic males and cisgender males (i.e., “men”) are often not perceived as being “homosexual” because they are hetero-gendered. An individual’s emic understanding of sexuality can be an important determinate of their sexual behavior and identity. Nevertheless, sexual behavior and sexual orientation identity (if one exists) are not necessarily concordant with an individual’s sexual orientation (e.g., [11], [12]). Bailey [13] describes sexual orientation in men as a mechanism, analogous to a compass, that directs sexuality and that reflects sexual feelings/arousal/fantasy/attraction rather than other factors such as social constraints. Implicit to this compass metaphor is the assumption that sexual orientation in men is oriented in one direction, as opposed to multiple directions. Hence, the question arises as to what the underlying sexual orientation is of the cisgender men who engage in sexual activity with feminine/transgender males, particularly in the numerous cultures where transgender male androphilia is the norm and sexual interactions between transgender androphilic males and cisgender men are relatively common.

In many respects, transgender males represent an amalgamation of both masculine and feminine traits to a relatively greater degree than cisgender males. For example, some transgendered males may be feminine in terms of their outward appearance but may nonetheless retain their male genitalia. This renders tenable the possibility that their cisgender male sexual partners are sexually attracted to both men and women. Indeed, many cisgender men who have sex with transgender androphilic males engage in sexual activity with women as well [14]. Consequently, it is possible that such men are *bisexual* with respect to their sexual orientation (i.e., substantially sexually attracted to both adult males and adult females). If, in those non-Western cultures were transgender male androphilia predominates, a substantial percentage of cisgender men were shown to be bisexual, this would stand in stark contrast to studies conducted in Western cultures, which suggest that male bisexual orientation is rare. For example, in Western settings relatively few men report substantial sexual feelings towards both men and women [15–18]. Similarly, in studies that employ measures of penile plethysmography, bisexual patterns of genital arousal have sometimes not been found, even among bisexually-identified men (e.g., [11], [12]).

Alternatively, it is possible that the cisgender men in question are truly *gynephilic* (i.e., sexually attracted to adult females), but they have sex with transgender androphilic males when they are unable to access adult women. Such a compromise may seem perplexing from a Western cultural perspective, however, in cultures where transgender male androphilia predominates, a substantial number of cisgender men may prefer sex with women whenever they are given the choice, but may nevertheless exhibit relatively little sexual aversion to the idea of engaging in certain types of same-sex sexual interactions with transgender males if women unavailable [14]. This may be because, to a certain extent, transgender androphilic males resemble their preferred sexual partners (i.e., adult women).

A third possibility is that some of the cisgender men who engage in sexual interactions with transgender androphilic males may be androphilic themselves, but not transgender. Masculine androphilic males can be described as *cisgender androphilic males* because their adult gender role expression more or less matches the gender they were assigned at birth. In short, the cisgender men who are the sexual partners of transgender androphilic males could be gynephlic, bisexual, or androphilic.

Although a considerable body of literature exists on transgender androphilic males, very little research has been conducted on their sexual partners—save for a more narrowly focused body of research on HIV contagion risk and prevention (e.g. [19–21]). There are a number of reasons why additional and more diverse research would be desirable. First, in cultures where transgender male androphilia predominates, our understanding of male-male sexuality will be partial, at best, until research is conducted on their cisgender male sexual partners. Second in many cultures, sexual interactions between transgender androphilic males and cisgender men may be a more ubiquitous feature of male sexuality than has previously been appreciated or acknowledged. Third, research indicates that the ancestral form of male androphilia was likely the transgender form [8]. Consequently, sexual interactions between transgender androphilic males and cisgender men were a likely feature of ancestral human mating systems and could have potentially influenced evolutionary processes such as sexual selection via *inter*-sexual mate competition between transgender males and women for cisgender male sexual partners [22]. Finally, there is currently debate in the sexology literature regarding the nature and prevalence of male bisexuality (cf. [11], [23–26]). Specifically, do self-identified bisexual men have a unique pattern of sexual attraction and arousal compared to men who self-identify as homosexual or heterosexual? Furthermore, what qualifies as a unique pattern of bisexual attraction and arousal? Cross-cultural research on the cisgender men who are sexual partners of transgender androphilic males could help to inform this debate.

In this study, we sought to characterize the sexual orientation of Samoan cisgender men who engage in sexual interactions with Samoan transgender androphilic males (known locally as *fa’afafine*) by assessing sexual preferences via measures of self-report and viewing time. Viewing time is measured by asking participants to subjectively rate the attractiveness of stimuli images while covertly recording their response time latencies (i.e., the amount of time elapsed between the presentation of the stimulus and participant response). It has been repeatedly demonstrated that heterosexual and homosexual men and women attend to images of their preferred sex for a longer period of time than their non-preferred sex, thus indicating that viewing time is a reliable assay of an individuals sexual orientation [27–30]. Furthermore, men who self-identify as bisexual exhibited response latencies to stimuli of men and women that were less dissociated from each other compared to those of both homosexual and heterosexual men [31–33]. In other words, bisexually identified men exhibited a unique “bisexual” pattern of response latencies. Viewing time measures have also been shown to correlate with physiological measures of sexual orientation such as pupil dilation [33], which have in turn been shown to correlate with genital arousal [34].

If the Samoan cisgender men who engage in sexual interactions with *fa’afafine* are sexually attracted to both men and women, then they should exhibit patterns of self-reported sexual attraction and response latencies to stimuli of both men and women that are less dissociated from each other compared to those of: (1) Samoan cisgender men who only engage in sexual interactions with women and (2) *fa’afafine*. Alternatively, if the cisgender men who engage in sexual interactions with *fa’afafine* are gynephilic, then they should exhibit patterns of self-reported sexual attraction and response latencies that are similar to those of cisgender men who only engage in sexual interactions with women. Finally, if the cisgender men who engage in sexual interactions with *fa’afafine* are androphilic, then they should exhibit patterns of self-reported sexual attraction and response latencies that are similar to those of *fa’afafine*.

## Methods

### Ethics Statement

This research was approved by the University of Lethbridge Human Subjects Research Ethics Committee. A Samoan Research Visa was obtained from Samoan Immigration under the auspices of the Samoan Ministry of Women, Community and Social Development. Participants were required to provide informed written consent prior to taking part in the study.

### Participants

All participants were recruited from across the island of Upolu, the most highly populated island of Independent Samoa, using a network sampling procedure, which involved contacting initial participants who displayed qualities of interest (i.e., status as [a] *fa’afafine*, [b] cisgender man who engages in sexual interactions with women exclusively, or [c] cisgender man who engages in sexual interactions with *fa’afafine*) then obtaining referrals from them to additional participants who, in turn, provided further referrals, and so on. All *fa’afafine* participants self-identified as such, only engaged in sexual interactions with men, and had done so within the past year (*N* = 21). Participants who self-identified as “men” were categorized as “men who engaged in sexual interactions only with women” if they engaged in sexual interactions exclusively with women throughout their lives, and had done so during the past year (*N* = 27). Participants who self-identified as “men” were categorized as “men who engaged in sexual interactions with *fa’afafine*” if they engaged in sexual interactions with *fa’afafine* throughout their lives, and had done so during the past year (*N* = 35). Men who engage in sexual interactions with *fa’afafine* varied in terms of their sexual partner profiles. For example, these men could engage in sexual interactions: (1) only with *fa’afafine*, (2) with *fa’afafine* and women, (3) with *fa’afafine* and men or (4) with *fa’afafine*, women and men. Table 1 contains information pertaining to the percentage of participants who fit into each of these groups relative to their entire lifespan and, more narrowly, in terms of the past year. The majority of participants in this group had engaged in sexual interactions with fa’afafine and women, but not men throughout their lives (60%; *n* = 21), and within the past year (74.3%: *n* = 26).

**Table 1:** Sexual partner profiles of men who engage in sexual interactions with fa’afafine.

Number of Participants	Precent of Sample Category	*Gender category of individuals with whom participants have engaged with sexually*		
	(%)	Men	Women	*Fa’afafine*
Throughout their lives:				
(*n* = 8)	22.9	✓	✓	✓
(*n* = 21)	60		✓	✓
(*n* = 4)	11.4	✓		✓
(*n* = 2)	5.7			✓
Within the past year:				
(*n* = 3)	8.6	✓	✓	✓
(*n* = 26)	74.3		✓	✓
(*n* = 2)	5.7	✓		✓
(*n* = 4)	11.4			✓

The age range of the *fa’afafine* participants was 19–43 (*M* = 29, *SD* = 7.06), that of men who engage in sexual interactions only with women was 20–46 (*M* = 30.44, *SD* = 8.95), and that of men who engage in sexual interactions with *fa’afafine* was 20–42 (*M* = 25.03, *SD* = 5.06). A one-way analysis of variance (ANOVA) indicated that age differed significantly as a function of group, *F* (2, 80) = 4.94, *p* = .009. Further analyses indicated that age was significantly correlated with length of response time to images of men by men who only engage in sexual interaction with women, *r* = .13, *p* = .009 and consequently, this was controlled for in subsequent viewing-time analyses. Age did not correlate significantly with self-reports of sexual attraction (range of *p* values = .088–.968) and consequently, was not controlled for in subsequent analysis of self-report. An independent chi-square test indicated religiosity (response options included: “not religious”, “somewhat religious”, “very religious”) did not differ significantly between groups, *χ* ^2^ (4) = 6.23, *p* = .183 (*fa’afafine*, men who only engage in sexual interactions with women, men who engage in sexual interactions with *fa’afafine*, respectively; highly religious: 23.8%, 33.3%, 8.6%; somewhat religious: 71.4%, 63.0%, 82.9%; not religious: 4.8%, 3.7%, 8.6%).

### Measures

The study consisted of a viewing-time experiment followed by a brief biographic questionnaire. The text accompanying the viewing-time experiment and questionnaire were translated and back-translated by two Samoan-speaking research assistants. One of the Samoan research assistants (a *fa’afafine*) was present to provide instructions to all of the participants and to answer questions.

Prior to the actual experiment beginning, participants first viewed nine trial images of men and women to familiarize them with the task. Because some participants were unfamiliar with computers, if they did not understand the experiment following the first trial, a second trial was conducted. If, following a third trial, the participants did not understand the task, they were given payment and thanked for their time. This resulted in disqualification of five potential participants. The experiment proceeded following one, two, or three practice trials, if (1) the participants stated they understood the task, and (2) were judged to have understood it by both the Samoan research assistant and the last author.

The viewing-time portion of the study was conducted using Empirisoft’s MediaLab viewing-time software. Participants were shown a series of images that included men’s faces, women’s faces, and neutral stimuli (i.e., neutral cartoon faces composed of a circle with two dots for eyes and a straight line for a mouth each of which varied slightly) and told that the purpose of the experiment was to obtain their subjective sexual attraction ratings for these images. Participants were instructed to take as long as they needed to complete the task and to carefully appraise each photo before rating it. Examples of the stimuli are displayed in S1 Appendix. The experiment consisted of forty images.

The first image in the actual experiment was a neutral cartoon face image. Participants’ response to this first neutral image was deleted from the analysis to remove any confounds associated with transitioning from the trial to the actual experiment. The remaining experiment was comprised of ten target images of women’s faces, ten target images of men’s face, and ten neutral cartoon face images, which were presented in a randomized order. As each image was displayed, participants were asked to respond to the question, which appeared at the top of the image: “How would you feel about having sex with this person?” Participants’ responses were measured using a seven point Likert-type scale ranging from 1-“very unpleasant” to 7- “very pleasant.” These response options appeared in a boxed column at the right of the image. Participants indicated their responses by clicking on the appropriate boxed number using a computer mouse.

Unbeknownst to the participants, as they were they providing their self-reported ratings of sexual attraction to the target images, the time between the presentation of the stimulus and participants’ response was being simultaneously recorded. It is important to note that this latent period, which is typically referred to as a “viewing time” reflects the time required to respond to the task of rating attraction (see [27], [35]). However, for ease of comparison across studies, we will refer to this measure as *viewing time*. Viewing times provided a measure of participants’ sexual attraction to the target images that is less subjective than self-report.

The Samoan research assistant was present during the trial portion of the viewing-time experiment, but left prior to the actual experiment commencing. The last author was present throughout the entire period of data collection for every participant. During the experiment he remained silent, did not move, did not look directly at the participants, and watched the computer screen out of the corner of his eye. The experiment was discontinued for any of the following non-exclusive reasons, including, if the participant: (1) looked away from the computer screen, (2) called out to someone, (3) lost control of the mouse, (4) moved rapidly through the images in a “machine-gun” fashion such that the last author inferred that they were not actually looking at the images but rather rushing to complete the experiment, (4) scored every one of the thirty-one experimental images the same, including the first neutral face image. This protocol resulted in incomplete viewing-time data from nine participants (3 *fa’afafine*, 3 men who engage in sexual interactions with *fa’afafine*, 3 men who only engage in sexual interactions with women), which was discarded.

Following the viewing-time experiment, the Samoan research assistant returned to help the participant complete the biographic questionnaire portion of the study. During the biographic questionnaire portion of the study, participants were asked to report their age, religiosity (“not religious”, “somewhat religious”, “very religious”) and whether they had had sexual interactions with men, women, and *fa’afafine* (1) at any point in their lives and (2) within the past year.

Upon completion of the questionnaire, participants were debriefed and invited to ask any questions they might have about the study. All participants, regardless of whether they completed the experiment or not, were thanked and given $20 Western Samoan Tala as a gift to compensate them for their time.

### Stimulus Construction

Twenty-four Samoan men (age range = 18–28 years, *M* = 22.04, *SD* = 2.71) and 24 Samoan women (age range = 18–27 years, *M* = 21.67, *SD* = 2.76) were photographed under standard lighting conditions posing with a neutral expression. Stimulus images were created using composite images of these Samoan male and female faces and the composite faces were then manipulated to render them more masculine or feminine. Prior to manipulating masculinity/femininity, twenty ‘base faces” (10 men, 10 women) were constructed. The base faces were composite average faces that were constructed from two individual facial photographs in line with previous methods [36–38]. Individual facial photographs were paired randomly from a pool of 40 face images (20 men, 20 women) that were, themselves, drawn randomly from the overall sample of Samoan men’s (*n* = 24) and women’s faces (*n* = 24). The composite base faces were then made symmetrical prior to being transformed on a sexual dimorphism dimension using the shape linear difference between a composite of 50 men and an equivalent composite of 50 young adult women, in line with previous methods [39]. Transforms represented 50% ± the difference between these two composites, resulting in twenty faces that were +50% of the shape of the relevant sex (10 masculinized faces of men, 10 feminized faces of women; S1 Appendix). Composite faces are representative of the average traits of the faces within them, reducing idiosyncratic differences between faces. By following this procedure, the faces of men were transformed to be more masculine and the faces of women were transformed to be more feminine. In doing so we ensured that the target images were clearly masculine or feminine, thereby eliminating any possibility that the images could have been viewed as androgynous.

### Data Analysis

*Mean self-reported sexual attraction* and *mean response time latencies* were calculated for participants’ response the target images of men, as well as the target images of women. To directly compare individual participants’ responses to the images of men versus the images of women, the discrepancy in their mean responses to both types of images were calculated. The discrepancies in *self-reported sexual attraction* and *response latencies* were calculated using the following formula: mean self-reported attraction rating (or response latency for images of men)—mean self-reported sexual attraction rating (or response latency for images of women) = discrepancy in self-reported sexual attraction ratings (or response latencies). A score greater than 0 indicated androphilic attraction; a score lower than 0 indicated gynephilic attraction.

### Statistical Analysis

Analysis was conducted using IBM SPSS Statistics version 22. Data pertaining to the present analysis is available online at: http://dx.doi.org/10.6084/m9.figshare.1245099. A one-way ANOVA, (with the alpha level set at *a* = .05) was conducted to examine whether the mean discrepancies in self-reported sexual attraction to each stimuli category (i.e., men and women) differed as a function of group. A one-way ANCOVA (with the alpha level set at *a* = .05) was conducted to examine whether the mean discrepancies in response latencies for each stimuli category (i.e., men and women) differed as a function of group, while controlling for age.

Following between-group analysis, within-group one sample *t*—tests were conducted to assess the extent to which participants’ self-reported sexual attraction and response latencies differed from a theoretically equal response to images of men and women (with the alpha level adjusted to *a* = .017 to maintain a Type I Error rate of *a* = .05 across multiple tests). To further characterize the precise pattern of sexual attraction exhibited by men who engage in sexual interactions with *fa’afafine*, additional independent sample *t*-tests were conducted to compare our two groups of cisgender men (i.e., those who only engaged in sexual interactions with women vs. those who engaged in sexual interactions with *fa’afafine*) for their self-reported sexual attraction and their response latencies. The alpha level set at *a* = .05 for these analyses.

Next, analyses were conducted to assess the possibility that participants were indiscriminately responding to all of the target images. Namely, within-group paired sample *t*-tests were conducted to assess whether participants differed in their self-reported sexual attraction ratings and response latencies to the neutral images, when compared to the target images of men and images of women (with the alpha level set at *a* = .008 to maintain a Type I Error rate of *a* = .05 across multiple tests).

Finally, to examine the possibility that men who engage in sexual interactions with *fa’afafine* are composed of a mixture of androphilic and gynephilic men a Shapiro-Wilk test of normality was conducted (with the alpha level set at *a* = .05).

## Results

Mean and standard deviation values for participants’ self-reported ratings of sexual attraction and viewing times response latencies are displayed in Table 2 by group.

**Table 2:** Mean (± SD) values by group for self-reported sexual attraction ratings and response latencies (measured in milliseconds) for images of men, women, and neutral stimuli.

	*Fa’afafine*		Men who only engage in sexual interactions with women		Men who engage in sexual interactions with *fa’afafine*	
	*M*	SD	*M*	SD	*M*	SD
*Self-reported sexual attraction ratings to images of*:						
Women	1.21	.44	4.31	1.55	4.54	1.53
Men	5.36	1.43	1.09	.21	3.15	1.76
Neutral Stimuli	1.63	.92	1.39	.88	2.41	1.41
*Response latencies for images of*:						
Women	5768.83	5250.89	11513.23	9035.63	11629.39	7095.42
Men	6901.61	4134.63	5264.47	4296.02	10505.54	8322.69
Neutral Stimuli	5225.02	4708.11	5539.41	4891.02	8389.58	7105.29

### Self-Reported Sexual Attraction Analysis

**Fig 1:**
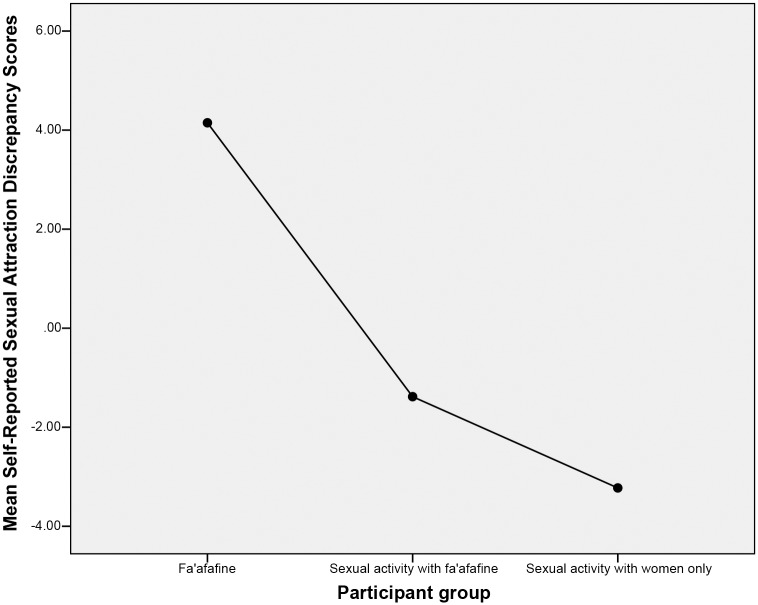
Mean Discrepancies in Self-Reported Sexual Attraction to Images of Men versus Images of Women for Fa’afafine, Men Who Engage in Sexual Interactions with Fa’afafine, and Men Who Engage in Sexual Interactions with Women Only.

Calculations of the discrepancies in self-reported sexual attraction to images of men and images of women revealed a mean score of *M* = 4.15, *SD* = 1.39 for *fa’afafine*; *M* = -3.23, *SD* = 1.55 for men who only engage in sexual interactions with women; and, *M* = -1.38, *SD* = 2.65 for men who engage in sexual interactions with *fa’afafine*. Group mean discrepancies in self-reported sexual attraction are displayed in Fig. 1. A one-way ANOVA was conducted to determine whether the mean self-reported sexual attraction discrepancy scores differed as a function of group. Because Leven’s test of homogeneity was significant, the Brown-Forsythe statistic is reported. This analysis indicated a significant main effect of group, *F* (2, 73.60) = 95.41, *p* <. 001, *ղ*_*p*_^2^ = .67. Post hoc analysis using Dunnett T3 indicated that the mean self-reported sexual attraction discrepancy score for *fa’afafine* was significantly higher than that of men who engage in sexual interactions with *fa’afafine* (*p* <. 001, Cohen’s *d* = 2.61), and men who only engaged in sexual interactions with women (*p* <. 001, Cohen’s *d* = 5). The mean self-reported sexual attraction discrepancy score for men who engage in sexual interactions with *fa’afafine* was significantly higher than that of men who only engage in sexual interactions with women (*p* = .003, Cohen’s *d* = .85).

Additional analyses were conducted to assess the extent to which groups differed from a pattern of equal sexual attraction the images of men and women, as measured by self-report. First, a within group one-sample *t*-test was conducted to assess whether groups differed significantly from a pattern of equal attraction to the images of men and women. A test value of 0 was used for all groups because this value indicates equal attraction to both men and women. The alpha level was adjusted to *a* = .017 to maintain a Type I Error rate of *a* = .05 across multiple comparisons. This analysis revealed that *fa’afafine* scored significantly higher than 0, *t* (20) = 13.69, *p* <. 001, Cohen’s *d* = 6.12; men who only engaged in sexual interactions with women scored significantly lower than 0, *t* (26) = -10.78, *p* <. 001, Cohen’s *d* = -4.23; and, men who engage in sexual interactions with *fa’afafine* also scored significantly lower than 0, *t* (34) = -3.09, *p* = .004, Cohen’s *d* = -1.06.

Addition analyses were conducted to further hone in on the precise pattern of sexual attraction exhibited by men who engage in sexual interactions with *fa’afafine*. An independent sample *t*-test indicated that men who engage in sexual interactions with *fa’afafine* differed significantly from men who only engage in sexual interactions with women in terms of their self-reported sexual attraction discrepancy scores, *t* (56.42) = 3.42, *p* = .001, Cohen’s *d* = .85.

### Viewing Time Analysis

**Fig 2:**
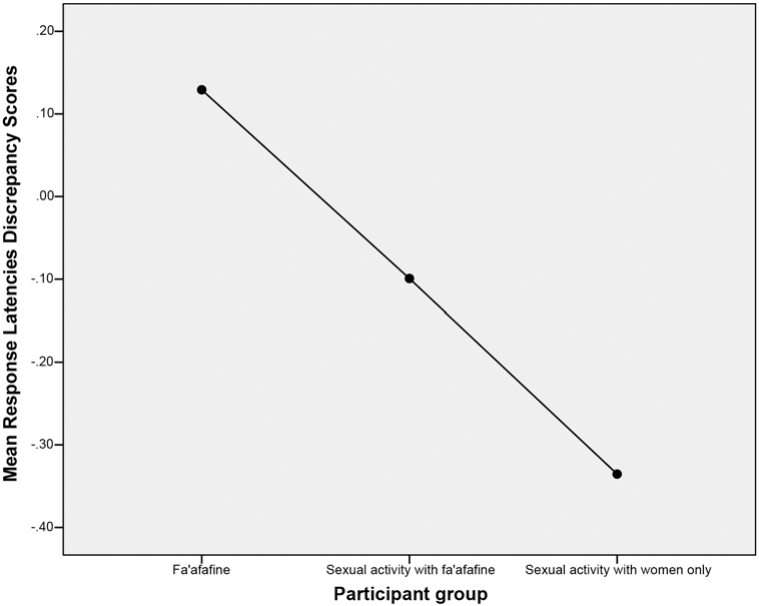
Mean Discrepancies in Response Latencies for Images of Men versus Images of Women for Fa’afafine, Men Who Engage in Sexual Interactions with Fa’afafine, and Men Who Engage in Sexual Interactions with Women Only.

A logarithmic transformation was conducted on the mean response latencies for images of women and men to ensure normality and avoid skew. Calculations of the mean response latencies discrepancy scores revealed a mean score of *M* = .12, *SD* = .17 for *fa’afafine*; *M* = -.35, *SD* = .20 for men who only engage in sexual interactions with women; and, *M* = -.09, *SD* = .16 for men who engage in sexual interactions with *fa’afafine*. Group mean discrepancies in response latencies are displayed in Fig. 2. An analysis of covariance (ANCOVA) was conducted, with age as a covariate, to determine whether mean response latencies discrepancy scores differed as a function of group. This analysis indicated a significant main effect of group, *F* (2, 79) = 42.12, *p* <. 001, *ղ*_*p*_^2^ = .52. There was no significant main effect of age, *F* (1, 79) = 2.91, *p* = .092, *ղ*_*p*_^2^ = .04. Post hoc pairwise comparisons, adjusted using Bonferroni correction, indicated that the mean response latencies discrepancy score for *fa’afafine* was significantly higher than that of men who engage in sexual interactions with *fa’afafine* (*p* <. 001, Cohen’s *d* = 1.26), and men who only engaged in sexual interactions with women (*p* <. 001, 2.56). The mean response latencies discrepancy score of men who engage in sexual interactions with *fa’afafine* was significantly higher than that of men who only engage in sexual interactions with women (*p* <. 001, Cohen’s *d* = 1.46).

Additional analyses were conducted to assess the extent to which groups differed from a pattern of equal sexual attraction to the images of men and women, as measured by viewing time. First, a within group one-sample *t*-test was conducted to assess whether groups differed significantly from a pattern of equal attraction to images of men and women. A test value of 0 was used for all groups because this value indicates equal attraction to both men and women. The alpha level was adjusted to *a* = .017 to maintain a Type I Error rate of *a* = .05 across multiple comparisons. This analysis revealed that *fa’afafine* scored significantly higher than 0, *t* (20) = 3.32, *p* = .003, Cohen’s *d* = 1.48; men who only engage in sexual interactions with women scored significantly lower than 0, *t* (26) = -9.17, *p* <. 001, -3.60; and, men who engage in sexual interactions with *fa’afafine* also scored significantly lower than 0, *t* (34) = -3.17, *p* = .003, Cohen’s *d* = -1.09.

Addition analyses were conducted to further hone in on the precise pattern of sexual attraction exhibited by men who engage in sexual interactions with *fa’afafine*. An independent sample *t*-test indicated that men who engage in sexual interactions with *fa’afafine* differed significantly from men who only engage in sexual interactions with women in terms of their response latencies discrepancy scores, *t* (60) = 5.76, *p* <. 001, Cohen’s *d* = 1.46.

### Responses to the Target Images and Neutral Control Images

Within group, paired sample *t*-tests were conducted to assess whether participants differed in their response to the neutral images compared to the target images of men and women, as measured by self-reported sexual attraction and viewing time. The alpha level was adjusted to *a* = .008 to maintain a Type I Error rate of *a* = .05 across multiple comparisons. Regarding self-reported sexual attraction, *fa’afafine* did not differ significantly in their ratings of the images of women and the neutral images, *t* (20) = -1.91, *p* = .071, Cohen’s *d* = -.58, but they did rate the images of men as significantly more attractive than the neutral images *t* (20) = 10.36, *p* <. 001, Cohen’s *d* = 3.1. Men who only engaged in sexual interactions with women did not differ significantly in their ratings of the images of men and the neutral images, *t* (26) = -1.85, *p* = .075, Cohen’s *d* = -.48, but they did rate the images of women as significantly more attractive than the neutral images, *t* (26) = 8.90, *p* <. 001, Cohen’s *d* = 2.31. Men who engage in sexual interactions with *fa’afafine* rated images of women as significantly more attractive than the neutral images, *t* (34) = 5.81, *p* <. 001, Cohen’s *d* = 1.44, but did not differ in their ratings of self-reported sexual attraction to the images of men and the neutral images given the adjusted alpha level, although the group differences trended towards significance in the expected direction, *t* (34) = 2.50, *p* = .017, Cohen’s *d* = .47.

With respect to viewing time, *fa’afafine* did not differ significantly in their response latency duration when presented with images of women and neutral images, *t* (20) = 1.93, *p* = .068, Cohen’s *d* = .22, but their response latencies were significantly longer when presented with images of men than when presented with neutral images, *t* (20) = 3.73, *p* = .001, Cohen’s *d* = .69. Men who only engage in sexual interactions with women did not differ significantly in their response latency duration when presented with images of men and the neutral images, *t* (26) = -.13, *p* = .899, Cohen’s *d* = -.02, but their response latencies were significantly longer when presented with images of women than when presented with neutral images, *t* (26) = 7.85, *p* <. 001, Cohen’s *d* = 1.17. The response latencies of men who engage in sexual interactions with *fa’afafine* were significantly longer when presented with images of women than when presented with neutral images, *t* (34) = 5.86, *p* <. 001, Cohen’s *d* = .69, and were significantly longer when presented with images of men than when presented with neutral images, *t* (34) = 3.33, *p* = .002, Cohen’s *d* = .35.

### Distribution of Responses to Images of Men and Women

If half of the men who engage in sexual interactions with *fa’afafine* were androphilic and the other half were gynephilic, then distribution frequencies for self-reported sexual attraction and response time latencies would be bimodal and mean sexual attraction scores for this group would mistakenly indicate a bisexual pattern of sexual attraction. To assess this possibility, we examined the extent to which our two measures of sexual attraction for men who engage in sexual interactions with *fa’afafine* conformed to a normal distribution, in which case, a bimodal pattern cannot be inferred. Distribution for self-reported sexual attraction discrepancy scores are displayed in S2 Appendix and response latencies discrepancy scores are displayed in S3 Appendix. A Shapiro-Wilk test of normality was conducted on self-reported sexual attraction and response latencies discrepancy scores for men who engage in sexual interactions with *fa’afafine*. For this analysis, mean discrepancy scores in participants’ response latencies for images of men and women, were calculated *without* logarithmically transforming the variables, so as not to impose normality on the scores. The mean and standard deviation discrepancy score for self-reported sexual attraction of men who engage in sexual interactions with *fa’afafine* was *M* = -1.38, *SD* = 2.65. This analysis obtained significance, *W* (35) = .932, *p* = .03, indicating that self-reported sexual attraction discrepancy scores of men who engage in sexual interactions with *fa’afafine* deviated from a normal distribution. In contrast, the mean and standard deviation discrepancy scores for response latencies of men who engage in sexual interactions with *fa’afafine* was *M* = -1123.85, *SD* = 3149.94. This analysis was not significant, *W* (35) = .983, *p* = .85, indicating that discrepancy scores for response latencies of men who engage in sexual interactions with *fa’afafine* did not deviate from a normal distribution.

## Discussion

The current study employed measures of self-reported sexual attraction and viewing time to determine whether Samoan cisgender men who engage in sexual interactions with *fa’afafine* exhibit a bisexual, gynephilic, or androphilic pattern of sexual attraction when compared to: (1) Samoan cisgender men who only engage in sexual interactions with women, and (2) *fa’afafine*. All groups differed from each other in their patterns of sexual attraction. Both self reported sexual attraction and viewing-time response latencies indicated that Samoan cisgender men who only engaged in sexual interactions with women exhibited a gynephilic pattern of sexual attraction, whereas *fa’afafine* exhibited an androphilic one. In comparison, Samoan cisgender men who engaged in sexual interactions with *fa’afafine* demonstrated a pattern of sexual attraction that was intermediate between, and significantly different from: (1) equal sexual attraction to the images of men and women, and (2) the more extreme pattern of gynephilic attraction exhibited by Samoan cisgender men who only engage in sexual interactions with women.

Both self-reported sexual attraction and viewing time measures employed in this study indicate that Samoan cisgender men who engaged in sexual interactions with *fa’afafine* exhibit: (1) significantly more sexual attraction to women than do *fa’afafine* and (2) significantly more sexual attraction to men then do Samoan cisgender men who only engage in sexual interactions with women. Consequently, on the basis of these measures and this sample, Samoan cisgender men who engage in sexual interactions with *fa’afafine* could be accurately described as exhibiting a bisexual pattern of sexual attraction. This bisexual pattern of sexual attraction was not characterized by perfectly equal sexual attraction to men and women but, it is important to note that such a theoretical ideal is rarely found in the real world [16].

If half the cisgender men who engage in sexual interactions with *fa’afafine* were composed of men who exhibit androphilic attraction, and the other half were composed of men who exhibit gynephilic attraction, then the resulting mean sexual attraction score would mistakenly indicate a pattern of bisexual attraction for this group. Analyses indicated that this type of bimodal group composition may account for the self-reported sexual attraction scores of the cisgender men who engage in sexual interactions with *fa’afafine*. However, similar analyses indicated that this type of bimodal group composition does not characterize the pattern of response latencies discrepancy scores of cisgender men who engage in sexual interactions with *fa’afafine*. Taken together, these findings suggest that our two measures of sexual attraction do not directly map onto each other for cisgender men who engage in sexual interactions with *fa’afafine*. Potential within-group variation exists in terms of these men’s subjective reports of sexual feelings. In contrast, more objective measures of sexual preference (i.e., response latencies discrepancy scores) indicate more within group uniformity.

*Fa’afafine* and men who only engage in sexual interactions with women had prolonged response latencies when presented with images of their preferred sex compared to neutral images. Men who engaged in sexual interactions with *fa’afafine* had prolonged response latencies for images of both men and women. Their response latencies for images of men versus neutral images were not significantly different given our adjusted alpha levels, although there was a clear trend towards significance. Absence of a significant effect may reflect Type II Error, and might disappear if a larger sample size is employed. Regardless, the tendency of these men to exhibit relatively similar viewing times for images of men and women can not be explained in terms of a general tendency to respond indiscriminately to all images, regardless of their content.

The bisexual pattern of viewing-time exhibited by Samoan men who engage in sexual activity with *fa’afafine* is similar to that which has been reported for bisexually-identified men in Canada [31] and the USA [32], [33]. When viewed from a comparative perspective, a number of insights can be drawn from these studies. First, because the category “bisexual” is not one that the vast majority of Samoan men draw upon to construct their identities, the manifestation of a bisexual pattern of viewing-time is not contingent on the existence of a bisexual identity. Second, men that exhibit bisexual viewing-times appear to engage in markedly different patterns of sexual behavior. In Canada, men who exhibit bisexual viewing-times report engaging in appreciable sexual activity with both men and women (e.g., [31] *M*, SD, number of male sexual partners: *47*.*4*, 153.6; number of female sexual partners, *14*.*1*, 13.2). However, in Samoa, fully 77.1% of the men who exhibited bisexual viewing times (i.e., men who engage in sexual interactions with *fa’afafine*) did not engage in sexual activity with both men and women; rather, these men reported engaging in sexual activity with just *fa’afafine* (7.4%), just *fa’afafine* and men (14.3%) or just *fa’afafine* and women (75%). While it is true that *fa’afafine* are male-bodied, they do not look or act like cisgender men. If we accept that bisexual viewing-times truly reflect patterns of sexual attraction then, on the basis of these studies, we must also accept that the manner in which bisexual patterns of sexual attraction manifest behaviorally vary from one culture to the next.

### Limitations

One potential limitation of the present study is our use of non-sexually suggestive stimuli. Traditionally viewing time studies have been conducted using more sexually suggestive stimuli, such as images of models in underwear or swimsuits (e.g., [28], [31], [32], [40]). However, due to Samoan cultural mores, it is uncommon for a woman to be seen in a swimsuit or otherwise minimally dressed, but it is unremarkable for a man to be seen in a similar state. Thus, using swimsuit or underwear clade models as stimuli in Samoa could introduce a potential confound because such imagery of women would be relatively novel, whereas, such imagery of men would be relatively commonplace. Furthermore, it is important to note that heterosexual gender difference in response latencies are maintained when only faces are used as stimuli [27]. In any case, one would anticipate that if the stimuli we employed were not adequately explicit our results would be biased toward Type II Errors (failing to reject a null hypothesis), which is inconsistent with our results due to our significant findings.

Additionally, to our knowledge, this study represents the first time a viewing time experiment pertaining to sexual orientation has been conducted in a non-Western field setting. Although every effort was made to ensure that all participants were tested under similar conditions, confounds may have been introduced due to variation in testing conditions. This limitation is somewhat mitigated, however, because this factor was true across all groups.

### Future Research Directions

Singer [41] conceptualized sexual arousal as consisting of three separate phases including: (1) an aesthetic phase that involves visual fixation on an object of interest, (2) an approach phase that involves desire to achieve closer physical proximity to an object of interest, and (3) a genital phase that involves various genital and non-genital physiological manifestations of sexual arousal. According to this model, genital arousal and pupil dilation studies measure Singer’s [41] genital (physiological) phase, whereas viewing-time studies measure the aesthetic phase. Viewing time measures of sexual attraction and pupil dilation measures of sexual arousal are highly correlated [33], as are pupil dilation and genital arousal measures [34]. Taken together, these studies suggest that that measures of viewing time may be a good proxy for measures of sexual arousal. If so, then one would expect that Samoan cisgender men who engage in sexual interactions with *fa’afafine* would exhibit bisexual patterns of pupil dilation and genital arousal more frequently then Samoan cisgender men who only engage in sexual interactions with women. Further research will be needed to ascertain whether this is indeed the case and to understand the relationship between different measures of sexual orientation (e.g., viewing-time, pupil dilation, genital arousal, thermography, implicit association tests, self-report).

There is currently debate in the literature concerning whether measures of bisexuality reflect sexual *attraction* to both males and females or, alternatively, sexual attraction to one sex coupled with relatively little sexual *aversion* to the other (cf. [24], [25]). Presumably, if sexual *aversion* characterized participants’ responses to their least preferred sex, then their self-report and viewing time measures of sexual attraction would have been significantly lower for images of their least preferred sex when compared to neutral images. However, reactions toward these two types of stimuli were not different for either of the two groups that would serve as our controls (i.e., *fa’afafine* and cisgender men who only engaged in sexual interactions with women). As such, we question whether these two control groups exhibited any aversive response whatsoever to their least preferred sex. It is possible that images of men’s and women’s faces were not sufficiently intense enough to elicit such a reaction. Future studies might elect to use more sexually explicit stimuli, which might increase the chances of obtaining a measurable aversive reaction.

The current study focused on examining whether our participants exhibited gynephilic, androphilic or bisexual viewing time patterns, but the ways in which sexual orientation can be manifested are not limited to these three patterns. For example, given that *fa’afafine* represent a particular combination of masculine and feminine characteristics, it is possible that their cisgender male sexual partners are *gynandromorphophilic* (i.e., sexually attracted to behaviorally and/or anatomically feminine males [42]). Future research could ascertain whether this is indeed the case by modifying the methodology employed here to include *fa’afafine* stimuli.

As our results indicate, the sexual partner profiles of men who engage in sexual interactions with *fa’afafine* varied greatly. Over half of these men also engage in sexual activity with women (60%) and almost a quarter also engaged in sexual activity with both men and women (22.9%). In addition, a substantial percentage of these men engaged in sexual interactions with *fa’afafine* and men, but not women (11.4%) and a small percentage engaged in exclusive sexual interactions with *fa’afafine* (5.7%). It would be informative if future research examined whether these men, who differ in their sexual partner profiles, also differ in terms of their self-reported sexual attraction, viewing times, and other measures of sexual orientation. The distribution of this group’s self-reported sexual attraction ratings of men and women indicate within-group variation, suggesting that this may indeed be the case. Furthermore, it would be of interest to examine whether differences in sexual activity profiles (e.g., willingness or unwillingness to engage in receptive/active fellatio and/or anal intercourse) exist among men who engage in sexual interactions with *fa’afafine* and, if so, whether these are related to differences in self-reported sexual attraction, viewing time, and other measures of sexual orientation.

The present study underscores the importance of conducting sexuality research outside of Western cultures if we seek to accurately characterize human sexual orientation (for a more general discussion of this point see [43]). Although the current study was not designed to provide an estimate of the frequency of male bisexuality in the Samoan population it is our strong impression, based upon ease of recruitment, that men who engage in sexual interactions with *fa’afafine* are commonplace. Indeed, most participants, including men who only sleep with women, indicated that this was the case. If so, and if these men can be accurately characterized as bisexual, then this would stand in stark contrast to the situation in Western cultures where male bisexuality is reported to be relatively rare [15–18]. Future research should focus on determining the prevalence of male bisexuality in Samoa using a probability sample.

## Supporting Information

S1 Appendix Examples of stimuli used in the viewing time experiment. A, Composite Images of Men. B, Composite Images of Women. C, Neutral Images.
(DOCX)

S2 Appendix Distribution of Self-Reported Sexual Attraction Discrepancy Scores for Images of Men and Images of Women for Men Who Engage in Sexual Interactions with Fa’afafine. (TIF)

S3 Appendix Distribution of Response Latencies Discrepancy Scores for Images of Men and Images of Women for Men Who Engage in Sexual Interactions with Fa’afafine. (TIF)
