# Supplementary material for: Viewing Time Measures of Sexual Orientation in Samoan Cisgender Men Who Engage in Sexual Interactions with Fa’afafine
Source: PLoS One. 2015 Feb 13;10(2):e0116529. doi: 10.1371/journal.pone.0116529 (PMC4332507; doi:10.1371/journal.pone.0116529)
Supplement: S1 Appendix — A, Composite Images of Men. B, Composite Images of Women. C, Neutral Images. (DOCX) [file pone.0116529.s001.docx]

**Supporting Information**

Appendix S1

*Examples of stimuli used in the viewing time experiment.*

A. Composite image of a man

**
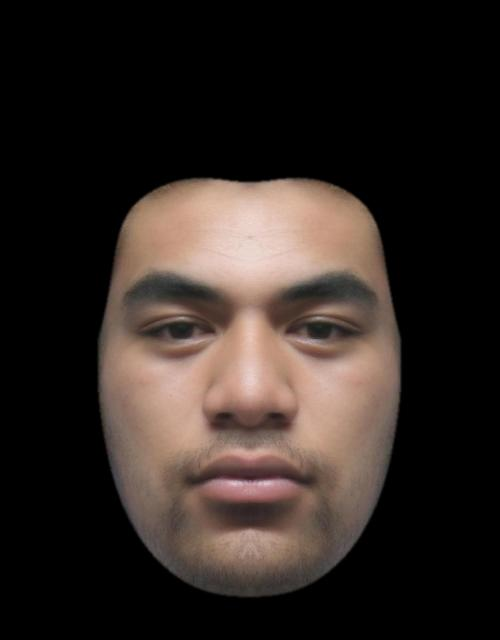
**

B. Composite image of a women

**
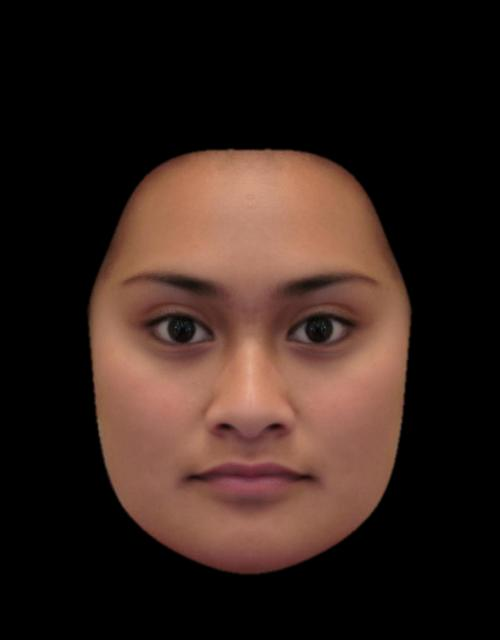
**

C. Neutral Image

**
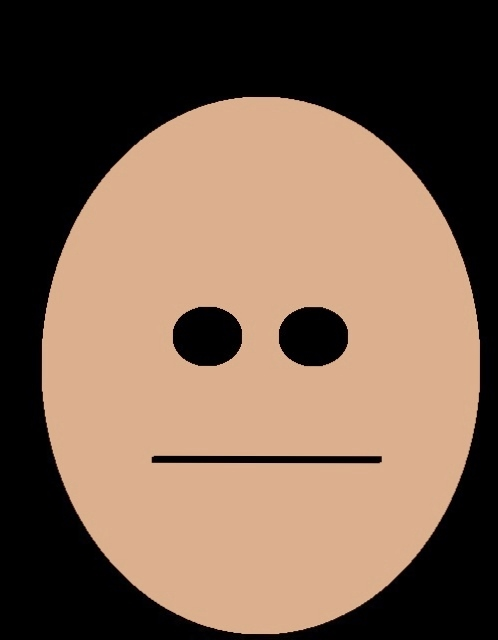
**
