# Supplementary figures and images for: Viewing Time Measures of Sexual Orientation in Samoan Cisgender Men Who Engage in Sexual Interactions with Fa’afafine
Source: PLoS One. 2015 Feb 13;10(2):e0116529. doi: 10.1371/journal.pone.0116529 (PMC4332507; doi:10.1371/journal.pone.0116529)

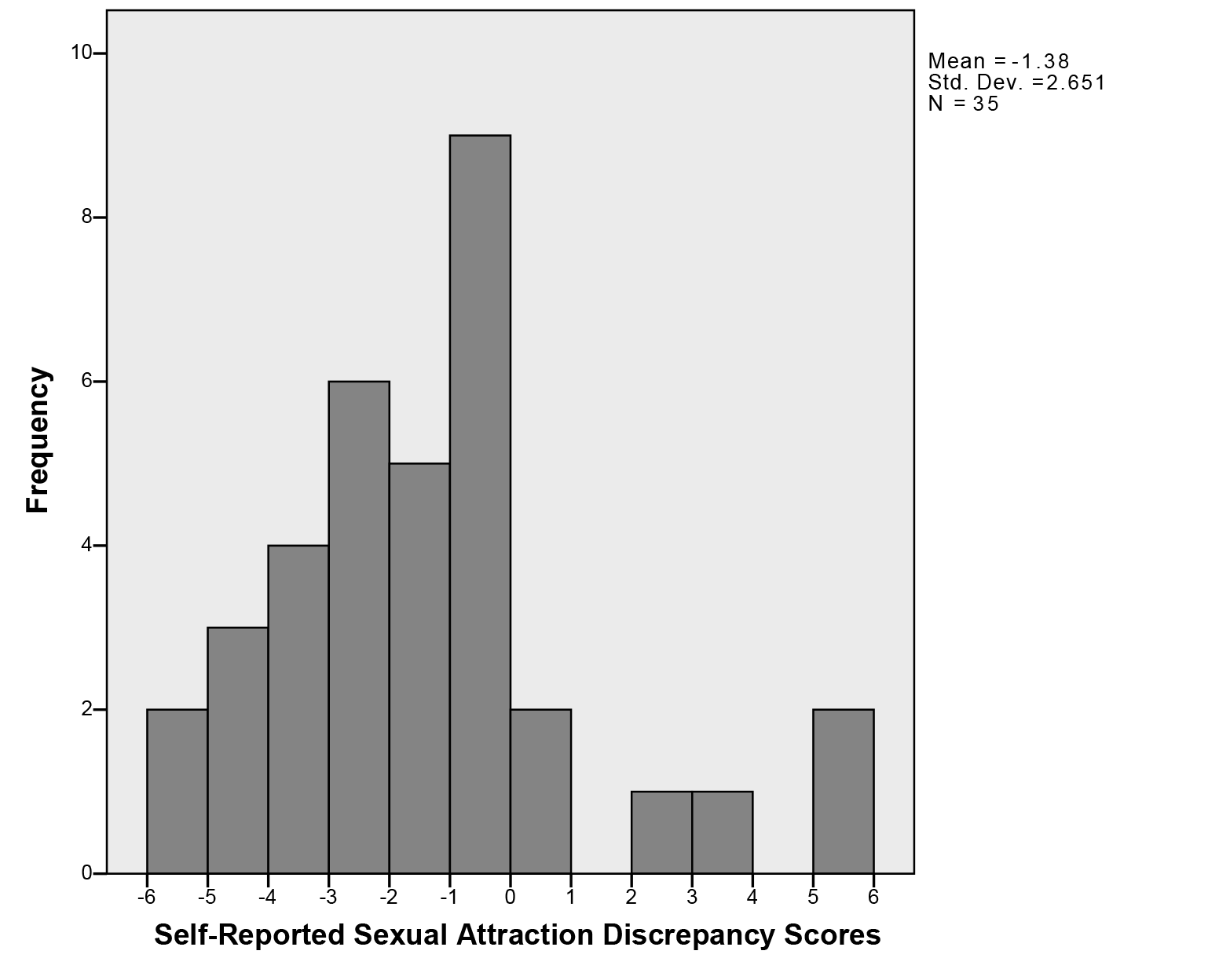

Supplement: S2 Appendix — (TIF) [file pone.0116529.s002.tif]

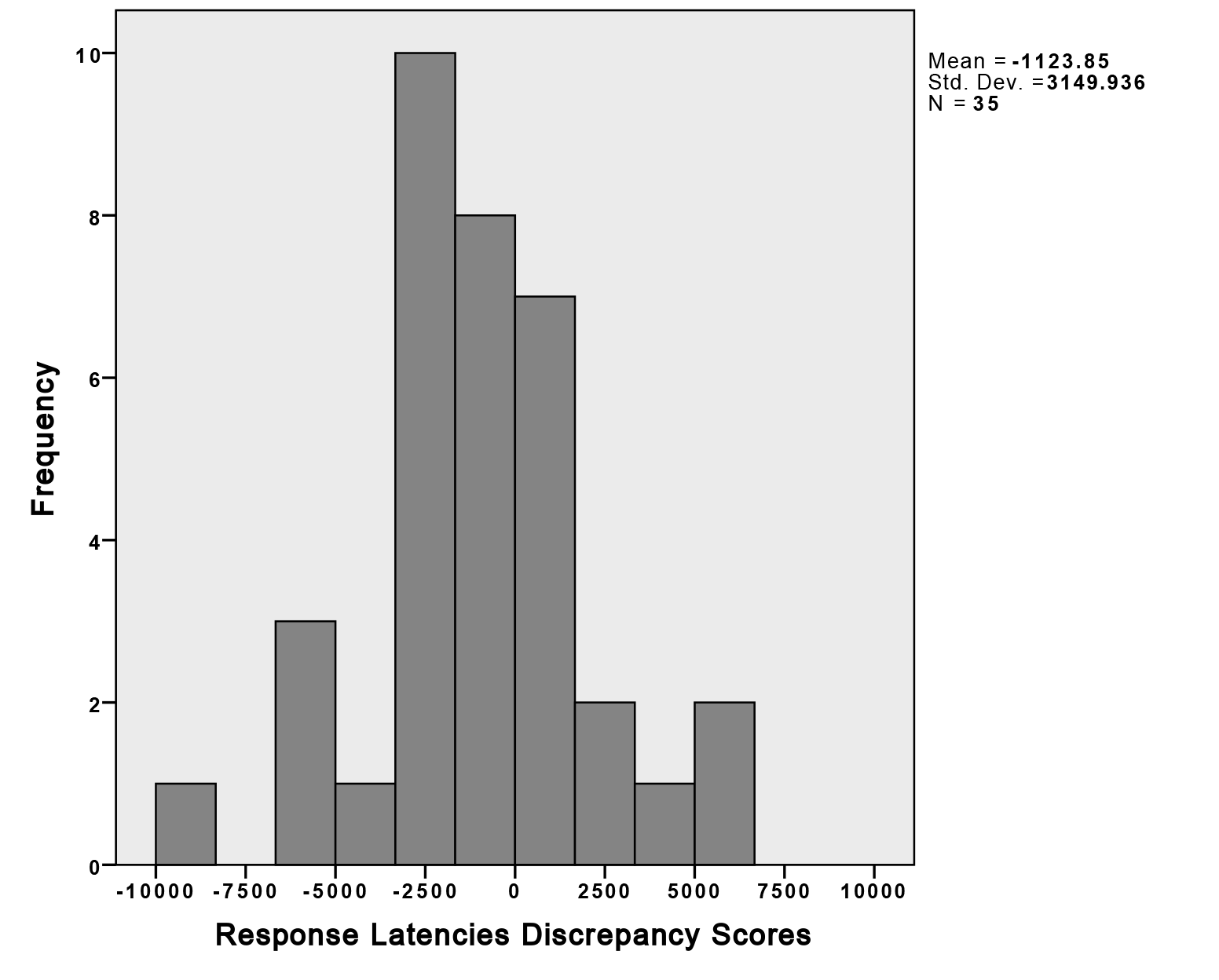

Supplement: S3 Appendix — (TIF) [file pone.0116529.s003.tif]
